# Rapidly Separable Micropillar Integrated Dissolving Microneedles

**DOI:** 10.3390/pharmaceutics12060581

**Published:** 2020-06-23

**Authors:** Chung-ryong Jung, Shayan Fakhraei Lahiji, Youseong Kim, Hyeonjun Kim, Hyungil Jung

**Affiliations:** 1 Nanobiotechnology Laboratory, Building 123, Yonsei University, 50 Yonsei-ro, Seodaemun-gu, Seoul 03722, Korea; yongie2@daum.net (C.-r.J.); lahiji@yonsei.ac.kr (S.F.L.); ustarkim@naver.com (Y.K.); 2 Juvic Biotech, Inc., No. 208, Digital-ro 272, Guro-gu, Seoul 08389, Korea; hjkim@juvicbio.com

**Keywords:** biodegradable microneedle, separable microneedle, transdermal drug delivery, micropillar integrated microneedle, non-invasive micropillar

## Abstract

Dissolving microneedle (DMN) patches were developed as efficient and patient-friendly transdermal delivery systems for biopharmaceuticals. However, recent studies have confirmed that the efficiency of DMNs to deliver biopharmaceuticals is highly reduced because of incomplete insertion caused by the stiffness and elastic properties of the skin. Therefore, micropillar integrated DMNs were developed to overcome the insertion limitations of DMN patches. Although micropillars were designed as integrated applicators to implant DMNs across the skin, they can also become inserted into the skin, leading to skin injury and inflammation. Herein, we have developed a separable micropillar integrated DMN (SPDMN) capable of inserting DMNs across the skin with high efficiency while minimizing skin injury risk through the introduction of a safety ring feature. Unlike previously developed systems, the SPDMN does not require continuous skin attachment and can be detached immediately post-application, leaving DMNs implanted inside the skin. Altogether, the findings of this study lead to the development of a quick, safe, and efficient DMN-based drug delivery platform.

## 1. Introduction

Skin, the largest organ of the human body, is a potential target for the delivery of biopharmaceuticals [1]. However, due to the barrier properties of the stratum corneum of the skin, the transdermal delivery of biopharmaceuticals is limited to low molecular weight lipophilic compounds [2,3]. Therefore, oral administration and hypodermic injection are among the most widely used drug delivery systems. Although the oral administration of biopharmaceuticals is patient-friendly, there is a risk of drug activity reduction due to the first pass metabolism effect of the gastrointestinal tract and gastric acids [4,5]. On the other hand, although hypodermic injection is capable of delivering drugs directly to the target site without affecting their activity, hypodermic injection is also not regarded as being an ideal drug delivery method because of the pain and infection risk associated with the needles [6]. To address the above-mentioned limitations, various transdermal delivery systems have been developed to improve the low transdermal permeation properties of the delivery systems [7]. Among these systems, dissolving microneedles (DMNs) have been introduced as patient-friendly systems that enable the successful delivery of the encapsulated biopharmaceuticals, regardless of their molecular weight, across the skin barrier [8,9]. In brief, DMNs are micro-dimensioned needles composed of a biodegradable polymeric matrix backbone and are capable of piercing the stratum corneum and undergoing dissolution in a minimally invasive manner [10,11].

DMNs are generally fabricated in the form of arrays on sticky patches, which enables their application to the skin [12]. However, because of the stiffness and elasticity of the dermis, there is a high risk of incomplete DMN insertion, leading to low delivery efficiency of the encapsulated compounds [13,14]. On the other hand, depending on the chemical structures and concentrations of backbone matrices used in the fabrication of DMNs, the dissolution time varies from minutes to hours, leading to inconvenience, irritation, and allergic reactions in some patients [15]. Therefore, various innovative systems, including double-layered and tip-loaded DMNs, arrow-head DMNs, physical and chemical application enhancers, and DMN applicators, have been developed to overcome the current patch-based DMN limitations [16,17,18,19,20,21,22]. However, double layer and tip-loaded DMNs are limited by low loading capacity and complexities that limit their use as an optimal platform for DMNs [23,24,25,26].

Recent publications using micro-molding methods and 3D printing technologies suggest the potential of fabricating separable DMNs [27,28,29]. In addition, micropillar integrated DMNs were introduced as systems capable of completely inserting DMNs into the skin [30,31]. By fabricating DMNs over micropillars, the incomplete skin insertion risk associated with the previously developed patch DMNs is reduced. However, as the diameter of micropillars is similar to or less than that of the DMN base, there is a risk of accidental insertion of the micropillar into the skin, leading to skin injury. It is important to take into consideration that insertion of DMN itself into the skin breaches the stratum corneum, resulting in innate immune response as well as potential risk of skin inflammation. Moreover, because of the strong adhesion force between DMNs and micropillars, the system must be attached to the skin surface throughout the dissolution process. Therefore, although micropillar integrated DMNs can achieve high delivery efficiency of the encapsulated biopharmaceuticals, the potential risk of skin damage and requirement of continuous attachment limits their use as an ideal platform.

In this study, to overcome the limitations associated with micropillar integrated DMNs, we developed rapidly separable micropillar integrated DMNs (SPDMN) that minimize the skin damage. SPDMNs, as they apply DMNs within a short time, can be used as a carrier for a wide range of drugs as well as a transdermal drug delivery tool for pediatric application as well [32,33]. To reduce the potential risk of skin damage, the micropillars were fabricated with a diameter of 500 μm and a curved edge. The base diameter of the DMNs was set at 300 μm, creating a 200 μm safety ring over each micropillar, ensuring that only DMNs were inserted into the skin. Moreover, the adhesion force of DMNs and micropillars in the newly developed SPDMNs was optimized in such a way that DMNs penetrated the skin and separated by applying a lateral force. This prevents the need for continuous attachment to the skin, reducing the risk of irritation and skin allergy. Through a series of in vitro evaluations, we examined the fracture, insertion, and separation forces of three geometries of micropillars, as well as the friction micropillars on the skin during the separation process. Overall, the results suggest that the risk of skin injury is minimized in SPDMNs fabricated with a micropillar diameter of 500 μm in comparison to micropillars with a smaller diameter and those without a safety ring or curved edge.

## 2. Materials and Methods

### 2.1. Development of Micropillar Arrays

On each plate, 3 × 3 micropillar arrays with 300-μm-tall polymethyl methacrylate (Sigma Aldrich, St. Louis, MO, USA) were fabricated as the base structure. Micropillars were fabricated through molding technique by developing a metal-based female mold and filling it with polymethyl methacrylate. Three types of micropillars with the following geometries were developed: (i) a 300 μm micropillar, (ii) 500 μm micropillar, and (iii) 500 μm micropillar with a 45° curved edge (Figure S1). The pitch of the micropillars was fixed at 1.5 mm.

### 2.2. Fabrication of SPDMNs

To fabricate SPDMNs, DMNs with a total height of 500 ± 63 μm and a base diameter of 300 ± 21 μm were fabricated over the micropillars. Dimensions were calculated based on evaluating geometries of 5 DMNs per group. Hyaluronic acid (HA; 32 kDa, Soliance, Pomacle, France) was used as the backbone polymer matrix of the DMNs and Rhodamine B (Sigma Aldrich) was employed as the drug surrogate. Briefly, 0.3% (*w*/*v*) of Rhodamine B was dissolved in distilled water and homogenized with 60% (*w*/*v*) of HA at a g-force of 335× g for 10 min using a centrifugal mixer (ARV-310; Thinky Corp., Tokyo, Japan). The mixture was then dispensed twice per micropillar using automated X, Y, and Z stages (SHOT mini 100-s, Musashi, Tokyo, Japan) and placed in a customized centrifuge to fabricate SPDMNs using the centrifugal lithography method at a g-force of 4,089× g with an acceleration and deceleration of 9 and 3, respectively [34].

### 2.3. Fracture and Separation Force Analysis

Vertical mechanical fracture force (*n* = 10/group) and separation force (*n* = 9/group) analyses of SPDMNs were performed using a Z0.5TN force analyzer (Zwick/Roell, Ulm, Germany) with a resolution of 24 bit at a speed of 1 mm/min. To measure the fracture force, SPDMNs were placed vertically against a sensor probe. The maximum measurable force was set at 1 N and the initial detected fracture was set as the fracture force of SPDMNs. The separation force of the DMNs was measured by placing the SPDMNs horizontally against the sensor probe. The initial detected force was set as the separation force of the SPDMNs.

### 2.4. Skin Insertion and Separation Analysis of SPDMNs

SPDMNs were inserted into pig cadaver skin with a surface area of 2.5 cm^2^ and thickness of 1.0 ± 0.2 mm (Cronex, Seoul, South Korea) using the Z0.5TN force analyzer at 1 mm/min with a maximum force set at 11 N. The SPDMNs were then gently removed and the application spots were dyed with 1% trypan blue solution (*w*/*v*) for 10 min. The skin insertion success rate was measured by counting and analyzing the number of spots dyed by trypan blue (*n* = 5/group). The separation of SPDMNs was likewise confirmed upon applying a lateral force across the skin using a Z0.5TN at 1 mm/min. The SPDMN separation success rate was calculated based on the number of DMNs that detached from the micropillars post-insertion.

### 2.5. Skin Scratch Friction Force Analysis

The micropillars were positioned against the pig cadaver skin and moved sideways using the Z0.5TN force analyzer at a speed of 10 mm/min. To increase the contrast of the images of skin damage, they were converted into 8-bit images and processed using ImageJ software (National Institutes of Health, Bethesda, MD, USA). The force changes during the initial 0.8 mm displacement were recorded per micropillar. The average friction force of the micropillars against the skin was calculated based on the maximum fracture detected during each experiment (*n* = 5/group).

### 2.6. Statistical Analysis

Means were compared using the Student’s t-test or one-way analysis of variance (ANOVA) using the GraphPad Prism software (GraphPad Software, San Diego, CA, USA). *p*-values of <0.05 were considered significant.

## 3. Results and Discussion

### 3.1. Fabrication of SPDMNs

**Figure 1:**
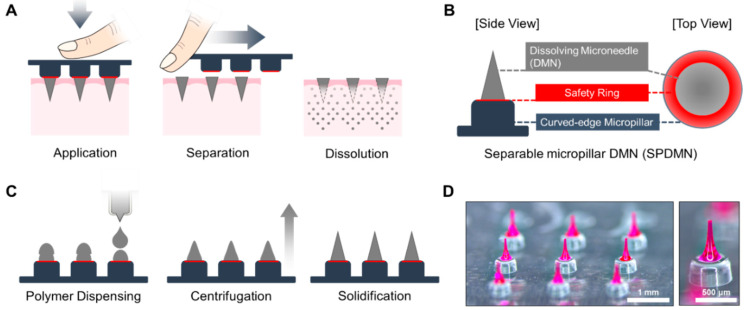
Application and characterization of SPDMNs. (**A**) Separation of DMNs from micropillars can be immediately achieved post-insertion through a lateral force. (**B**) A 200 μm safety ring surrounds the DMNs over the micropillars to ensure that only DMNs and not micropillars are inserted into the skin. The curved-edge characteristic of the micropillars is designed to minimize skin friction during the separation process. (**C**) The fabrication process of SPDMNs through centrifugation. (**D**) Actual microscope images of a 3 × 3 SPDMN array and a single SPDMN. Scale bars are 1 mm (left panel) and 500 μm (right panel).

SPDMNs were developed to improve the delivery efficiency of encapsulated compounds while minimizing the risk of skin irritation or allergic reactions by introducing the characteristic of immediate DMN separation from the micropillars (Figure 1A). Polymethyl methacrylate, as it is proved to not cause any irritation on the skin surface, was selected as the base material of micropillars [35]. To ensure that only DMNs and not micropillars were inserted into the skin, the base diameter of the DMNs was fabricated at 300 ± 21 μm, which was approximately 200 μm smaller than the base diameter of the 500 μm micropillars. The 200 μm safety ring surrounding the DMN acted as a protection layer for SPDMNs to minimize the risk of skin injury caused by the accidental insertion of micropillars into the skin (Figure 1B). Moreover, to separate DMNs from micropillars, the SPDMNs must be pushed laterally post-insertion over the skin surface. The resulting friction between the micropillars and skin may lead to skin injury and irritation. Therefore, to minimize accidental skin injury during the separation process, we introduced a 45° curve on the edges of the micropillars.

The fabrication process of the SPDMNs consisted of three main steps: (i) double dispensing of the polymer droplets over the micropillars, (ii) fabrication of DMNs over micropillars via the centrifugal lithography method, and (iii) solidification for 10 min (Figure 1C). Rhodamine B was used as a drug surrogate throughout the study to evaluate the skin penetration and separation efficiency of SPDMNs (Figure 1D). Although rhodamine B was employed as a drug surrogate for the proof-of-concept of SPDMN as a universal platform, the encapsulated compounds within DMNs can be selected within a wide range of micro- and macro-biopharmaceuticals based on the target disease [36].

### 3.2. Insertion and Separation Force Analysis

**Figure 2:**
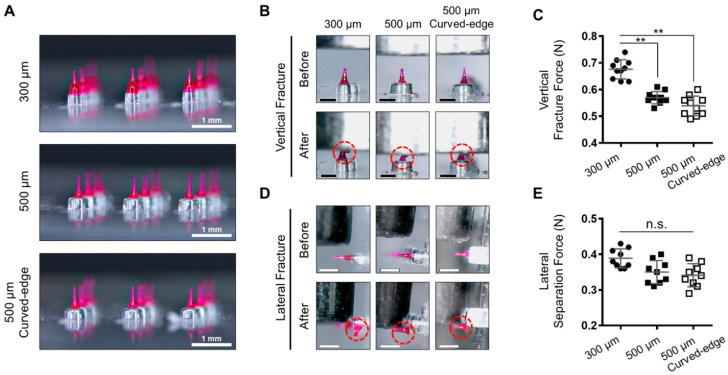
Fracture and separation force analysis. (**A**) DMNs were fabricated over 300 μm, 500 μm, and curved-edge 500 μm micropillars. (**B**) The process of fracture force assessment. (**C**) Mean fracture force of DMNs against the analyzer probe. (**D**) Separation force analysis through lateral probe analysis. (**E**) The lateral separation force of DMNs showed that less force was required to detach DMNs from both 500 μm micropillars. Dashed circles in (**B**,**D**) indicate DMNs post-analysis. Scale bars in (**A**) are 1 mm and in (**B**,**D**) are 500 μm. Data in (**C**,**E**) are expressed as mean ± S.D. **: *p* < 0.01. n.s.: not significant.

To evaluate the capability of SPDMNs in minimizing skin injury during the application process, three types of micropillars with different diameters (i) 300 μm, (ii) 500 μm, and (iii) 500 μm with a 45° curved edge were fabricated (Figure 2A, Figure S2). The heights of the micropillars and DMNs were fixed at 300 μm and 500 ± 63 μm, respectively. First, the vertical fracture force of DMNs was assessed to ensure that DMNs fabricated over micropillars had the required strength to penetrate skin without causing injury (Figure 2B). A single DMN per group was positioned vertically against a sensor probe, and its mechanical strength was evaluated. The results indicated that the maximum axial loads of DMNs fabricated over 300 μm, 500 μm, and curved-edge 500 μm micropillars were 0.67 ± 0.03 N, 0.57 ± 0.02 N, and 0.53 ± 0.3 N, respectively, which were considerably greater than the minimum force required to penetrate the skin [37,38] (Figure 2C).

Although DMNs in all groups were fabricated with the same volume of polymer, the vertical fracture force of DMNs fabricated over 300 μm micropillars was significantly higher than that of other DMNs. This is assumed to be due to the equal base diameters of DMNs and micropillars at 300 μm, resulting in a wider lower-portion geometry of DMNs than that of those fabricated over 500 μm micropillars (Figure S3). Next, to measure the minimum force required to separate DMNs from micropillars, each DMN was placed in a lateral position against the sensor probe of a fracture force analyzer (Figure 2D). DMNs fabricated over 300 μm, 500 μm, and curved-edge 500 μm micropillars were detached at minimum forces of 0.38 ± 0.02 N, 0.35 ± 0.03 N, and 0.34 ± 0.03 N, respectively (Figure 2E). These results suggested that the dimension of the micropillars does not impact the force required to separate the DMNs. Application of SPDMNs into the skin, however, is expected to exhibit a different separation characteristics. Although further detailed evaluations are currently underway, the force required to detach DMNs from micropillars in all three types has been confirmed by their ability to separate post-insertion into the skin. Furthermore, analyzing mechanical stability of DMNs through evaluating breaking and bending forces of DMNs would be helpful to fabricate optimal SPDMNs capable of separating DMNs with the least lateral force [39,40]. Compared with the conventional DMN patches and micropillars, which must be continuously attached throughout the DMN dissolution process, SPDMNs, due to their separation ability, are expected to reduce the risk of skin irritation.

### 3.3. Skin Application and Separation Assessment

**Figure 3:**
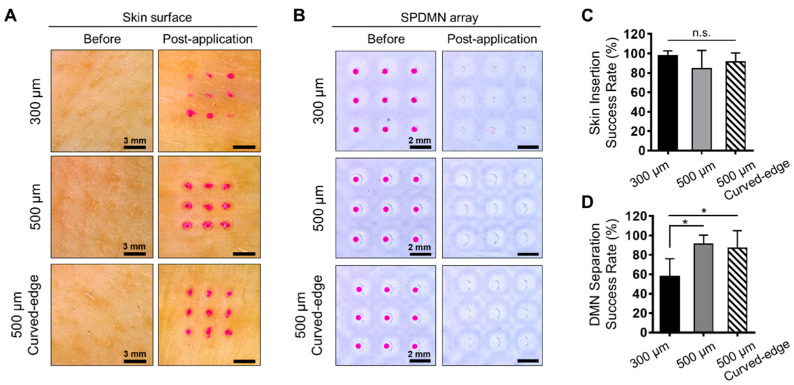
Application of SPDMNs into pig cadaver skin. (**A**) Application of DMNs fabricated over 300 μm, 500 μm, and curved-edge 500 μm micropillars into the skin. Application spots over the skin surface indicate successful separation of DMNs from micropillars. (**B**) Top-view images of SPDMNs pre- and post-application. DMNs were completely separated from micropillars post-insertion. (**C**) Comparison of skin insertion success rates. There were no significant differences between the insertion rates of SPDMNs. (**D**) The separation evaluation of SPDMNs confirmed significantly lower separation rates in 300 μm micropillars than in 500 μm and curved-edge 500 μm micropillars. Scale bars in panels of (**A**) is 3 mm and (**B**) is 2 mm. Data in (**C**,**D**) are expressed as mean ± S.D. *: *p* < 0.05. n.s.: not significant.

The adhesion force between DMNs and micropillars must be strong enough to insert them into the skin and weak enough to easily separate them upon application of lateral force. As confirmed through fracture and separation force analyses, single DMNs from each group had the required mechanical strength to penetrate the skin and were separated from micropillars by the application of lateral force. Therefore, to further confirm the results, we evaluated the application of DMNs fabricated over micropillars in 3 × 3 arrays using pig cadaver skin. In brief, SPDMNs were inserted into the skin using an automated force analyzer and immediately separated by applying lateral finger force toward the side. The results indicated that DMNs fabricated over 300 μm, 500 μm, and curved-edge 500 μm micropillars were successfully inserted into the skin (Figure 3A). Section analysis of pig cadaver skin at 10 min post application confirmed insertion of DMNs fabricated over micropillars into the skin (Figure S4). As pig cadaver skin exhibits dissimilar water content, the uneven over-diffusion of Rhodamine B over the skin surface in Figure 3A is natural [41,42,43]. Moreover, cadaver skins exhibit a lower skin elasticity and may be easier to be penetrated by DMNs. Therefore, further animal studies are required to confirm the skin insertion capability of SPDMNs in vivo. Next, the detachment of DMNs from micropillars was confirmed post-application (Figure 3B). DMNs fabricated over 500 μm and curved-edge 500 μm micropillars were successfully inserted into the skin and separated from the micropillars using lateral force. However, during the separation process, upon application of lateral force, some DMNs fabricated over 300 μm micropillars remained over the micropillars or were removed from the skin. This is assumed to be due to the difference in the lower portion geometry of the DMNs to those fabricated over 300 μm micropillars, which had wider lower portion geometry compared with those fabricated over 500 μm micropillars. This wider lower portion geometry may have led to the incomplete insertion of DMNs.

Next, to quantify the insertion characteristics of SPDMNs, the treated pig cadaver skins were dyed with trypan blue and the number of dyed spots over the skin were counted. The success rates of DMN insertions onto pig cadaver skin were 97.78 ± 4.9%, 84.44 ± 18.59%, and 91.11 ± 9.30% for 300 μm, 500 μm, and curved-edge 500 μm micropillars, respectively, with no significant differences (Figure 3C). Herein, the success rate was evaluated based on the number of pores created over the skin surface post-insertion and did not represent the successful application or dissolution of DMNs inside the skin. As SPDMNs were inserted into the skin through automated applicator, we achieved a high application success rate, whereas in actual application through finger force, there is a risk of accidental separation prior to insertion into the skin. The assessment of insertion characteristics and delivery efficiency of DMNs fabricated over micropillars remains to be addressed in our future studies.

Furthermore, the separation success rate was quantified based on the number of DMNs left over the micropillars post-insertion. Upon the application of lateral force, almost all DMNs were separated from the micropillars. However, some DMNs were strongly adhered to micropillars and were not immediately separated post-insertion. In 300 μm micropillars, the separation rates were significantly lower than those of 500 μm and curved-edge 500 μm micropillars at 55.77 ± 18.26%, 91.11 ± 9.30%, and 86.66 ± 18.26% (Figure 3D). Together, with the above insertion results, these findings confirmed that the fabrication of SPDMNs with the same diameter as that of the micropillars (300 μm) increased the potential risk of DMNs being removed from the skin. Altogether, we found that both 500 μm and curved-edge 500 μm micropillars could be considered ideal platforms to achieve the successful insertion and separation of DMNs.

Although further detailed investigations are required, we assume that increasing the insertion force, which was limited by maximum 11 N in this study, would increase the successful insertion rate and implant DMNs into deeper layers of the skin. However, this also increases the potential risk of skin injury caused by the accidental insertion of micropillars into the skin. Therefore, future studies regarding the correlation between the effects of insertion force and geometry of SPDMNs are required. Moreover, the impact of the safety ring on the penetration of DMNs into the skin remains to be addressed through section analysis.

### 3.4. Skin Friction and Damage Analysis

**Figure 4:**
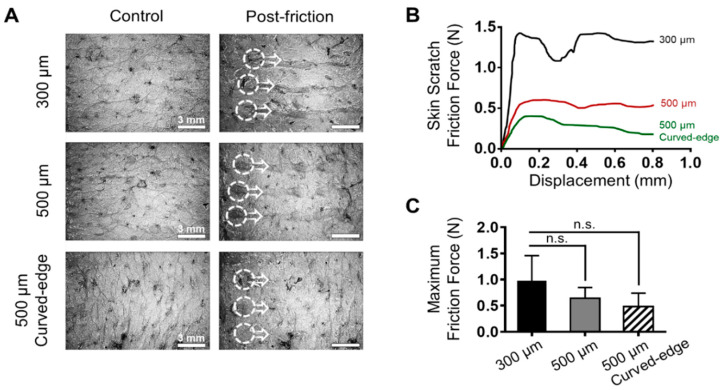
Evaluation of skin damage caused during the separation process of DMNs from micropillars. (**A**) Trace of micropillars over the skin surface upon application of the lateral force. The 300 μm micropillars caused considerable damage to the skin, whereas this was remarkably less in both 500 μm and curved-edge 500 μm micropillars. (**B**) Skin scratch friction force changes against displacement. The friction force of the 300 μm micropillars was higher than that of the other micropillars. (**C**) The maximum friction force of the micropillars against skin. Although 300 μm micropillars showed a higher friction force, due to stiffness properties of the skin, the data were not significant. Both 500 μm and curved-edge 500 μm micropillars had similar friction forces. Scale bars in (**A**) are 3 mm. Data in (**B**,**C**) are expressed as mean ± S.D.

Although the insertion of SPDMNs does not cause any significant skin surface damage, during the lateral separation process, as the micropillars slide over the skin surface they may scratch the skin. Therefore, to evaluate the impact of SPDMN geometry on skin injury during the application and separation processes, 300 μm, 500 μm, and curved-edge 500 μm micropillars were applied to the skin and the friction occurring during lateral force application was monitored (Figure 4A). Microscopic images of the skin after the separation process of SPDMNs showed that 300 μm micropillars caused a strong scratch over the skin. A considerably lower trace during lateral separation was seen in the 500 μm and curved-edge 500 μm micropillars than in the 300 μm micropillars. The smaller diameter of the 300 μm micropillars, as well as their sharp edges, are assumed to be responsible for the skin surface damage.

Next, to quantify the friction between the micropillars and skin, the lateral separation process was automatically performed using a force analyzer, and scratches up to 0.8 mm from the initial insertion point were monitored (Figure 4B). The friction of the 300 μm micropillars against the skin was remarkably higher than that of the 500 μm and curved-edge 500 μm micropillars, suggesting a lower risk of skin damage caused by 500 μm micropillars. The maximum force detected upon the friction between micropillars and the skin peaked at 0.1 mm from the initial separation point with 0.97 ± 0.47 N, 0.64 ± 0.20 N, and 0.48 ± 0.25 N in 300 μm, 500 μm, and curved-edge 500 μm micropillars, respectively (Figure 4C). Since the skin has dissimilar elasticity, water content, and stiffness properties, achieving a significant friction trend was not possible. Although the results were not significant, the maximum friction force applied onto skin by 300 μm micropillars was higher than that applied by both types of 500 μm micropillars. In addition, there were no remarkable differences between the friction forces of 500 μm and curved-edge 500 μm micropillars. These results indicate that DMNs fabricated over 500 μm micropillars can successfully minimize skin damage during the separation process. Overall, based on the fraction, detachment, and skin friction analyses, the curved-edge 500 μm micropillars were found to be the ideal platform for the rapid application of DMNs into skin. Combined with previous findings regarding the improved insertion accuracy and delivery efficiency of biopharmaceuticals encapsulated within micropillar integrated DMNs [13], this study suggests that the introduction of a curve to the edge of the micropillars would further increase their potential as universal platforms for the delivery of DMNs. Moreover, these findings suggest that using micropillars that have a wider diameter than the base area of DMNs, a safety ring in 500 μm micropillars, and curved edges not only prevents the accidental insertion of micropillars into the skin but also decreases skin friction. Although this study provides a fundamental investigation into the fabrication of an SPDMN platform to minimize skin damage, detailed evaluations regarding skin damage through in vivo evaluations and transepidermal water loss experiments remain to be addressed in future studies.

## 4. Conclusions

We demonstrated the fabrication of and evaluated an SPDMN transdermal delivery platform that effectively inserts DMNs into the skin, separates from DMNs upon application of a lateral force, and minimizes skin damage during the separation process. In 500 μm and curved-edge 500 μm micropillar-based SPDMNs, a safety ring surrounding the DMNs was designed to prevent the accidental insertion of micropillars into skin, remarkably improving their application safety. In brief, this study showed that the separation capability was higher in DMNs fabricated over 500 μm and curved-edge 500 μm micropillars (91.11 ± 9.30% and 86.66 ± 18.26%, respectively), than over 300 μm micropillars (55.77 ± 18.26%). Through a series of in vitro evaluations, we confirmed that the skin friction post-insertion was remarkably lower in DMNs fabricated over 500 μm and curved-edge 500 μm micropillars (0.64 ± 0.20 N and 0.48 ± 0.25 N, respectively), than over 300 μm micropillars (0.97 ± 0.47 N), suggesting their potential as patient-friendly transdermal delivery platforms for pharmaceuticals.

## Supplementary Materials

The following are available online at https://www.mdpi.com/1999-4923/12/6/581/s1, Figure S1. The micropillars were fabricated at a total height of 300 μm with base diameters of 300 μm (top panels) and 500 μm (middle panels). In addition, a 500 μm with a 45° curved edge was fabricated to evaluate the damage impact of micropillar edge on the skin (bottom panels). The 3 × 3 array of micropillars were fabricated with a fixed pitch of 1.5 mm. The scale bars in all panels are 1 mm; Figure S2. Specification of micropillars. The height of DMN and micropillars in all three types were fixed at 500 ± 63 μm and 300 μm, respectively. (A) The base diameter of miropillar and DMN were at 300 μm. (B) The base diameter of micropillar was 500 μm, which is 200 μm larger than the base diameter of DMNs. (C) A 45° curve was introduced to the edge of micropillars to minimize the friction force during SPDMN separation process; Figure S3. Geometry of DMNs fabricated over the micropillars. The DMNs fabricated over 300 μm-micropillars had a wider mid-portion compared with those fabricated over the 500 μm-micropillars; Figure S4. (A) Section analysis of pig cadaver skin at 10 min post application of single SPDMNs for 300 μm, 500 μm and 500 μm curved-edge respectively. Yellow color (highest intensity) indicates the DMN application site and the red color shows permeation of rhodamine inside the skin. Dashed line indicates the skin surface. (B) Arrayed section analysis of pig cadaver skin at 10 min post application of 500 μm curved-edge SPDMN.
