# Supplementary material for: Rapidly Separable Micropillar Integrated Dissolving Microneedles
Source: Pharmaceutics. 2020 Jun 23;12(6):581. doi: 10.3390/pharmaceutics12060581 (PMC7356013; doi:10.3390/pharmaceutics12060581)
Supplement: Supplementary file 1 [file pharmaceutics-12-00581-s001.pdf]

pharmaceutics

Supplementary Materials: Rapidly separable micropillar integrated dissolving microneedles

Chung-ryong Jung, Shayan Fakhraei Lahiji, Youseong Kim, Hyeonjun Kim and Hyungil Jung

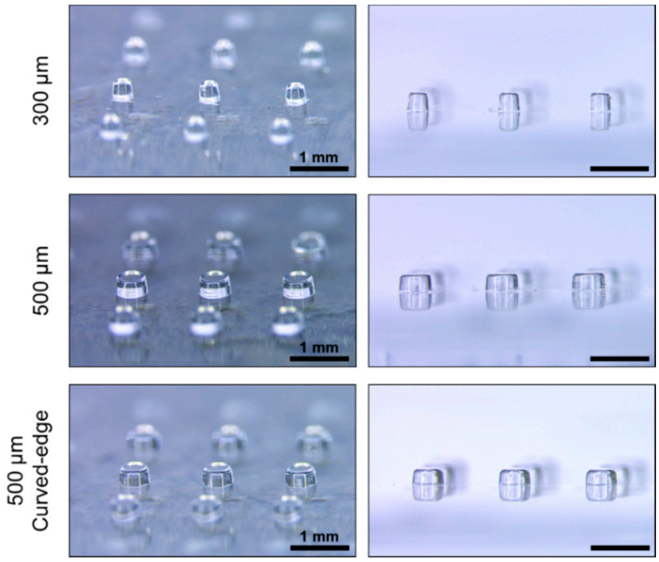

Figure S1. The micropillars were fabricated at a total height of 300 µm with base diameters of 300 µm (top panels) and 500 µm (middle panels). In addition, a 500 µm with a 45° curved edge was fabricated to evaluate the damage impact of micropillar edge on the skin (bottom panels). The 3 × 3 array of micropillars were fabricated with a fixed pitch of 1.5 mm. The scale bars in all panels are 1 mm.

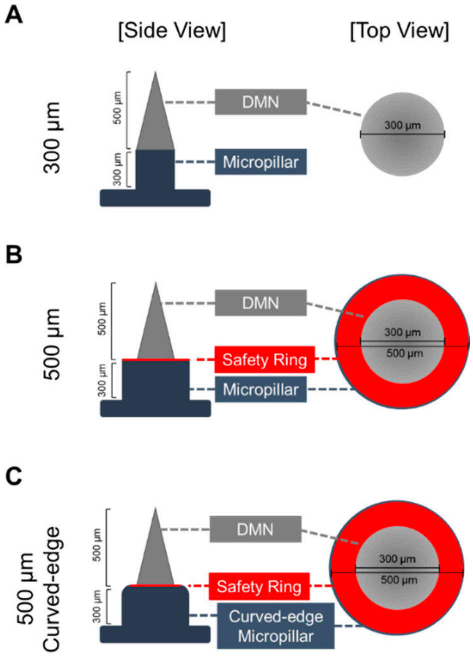

Figure S2. Specification of micropillars. The height of DMN and micropillars in all three types were fixed at 500 ± 63 µm and 300 µm, respectively. (A) The base diameter of miropillar and DMN were at 300 µm. (B) The base diameter of micropillar was 500 µm, which is 200 µm larger than the base diameter of DMNs. (C) A 45° curve was introduced to the edge of micropillars to minimize the friction force during SPDMN separation process.

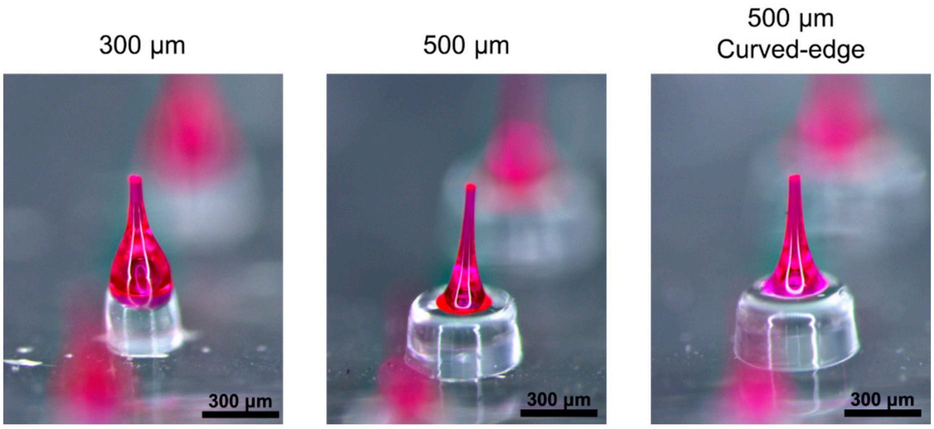

Figure S3. Geometry of DMNs fabricated over the micropillars. The DMNs fabricated over 300 µm-micropillars had a wider mid-portion compared with those fabricated over the 500 µm-micropillars.

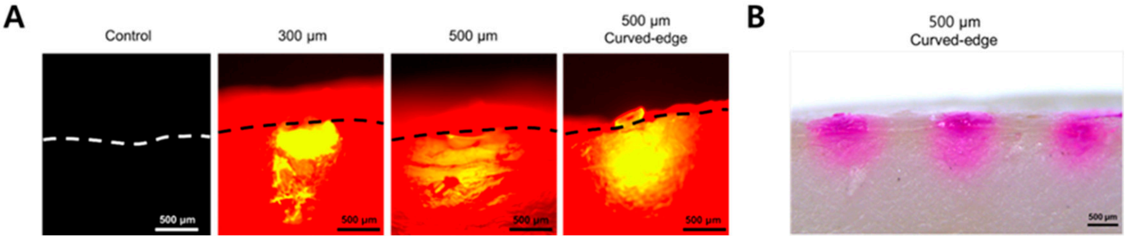

Figure S4. (A) Section analysis of pig cadaver skin at 10 min post application of single SPDMN for 300 µm, 500 µm and 500 µm curved-edge, respectively. Yellow color (highest intensity) indicates the DMN application site and the red color shows permeation of rhodamine inside the skin. Dashed line indicates the skin surface. (B) Arrayed section analysis of pig cadaver skin at 10 min post application of 500 µm curved-edge SPDMN.
